# Support from Advisors on Child Rearing for Alleviating Maternal Anxiety and Depressive Symptoms among Japanese Women

**DOI:** 10.2188/jea.JE2007456

**Published:** 2008-10-01

**Authors:** Yuki Sato, Tadaaki Kato, Naoko Kakee

**Affiliations:** 1 Department of Health Policy, National Research Institute for Child Health and Development.

**Keywords:** Social Support, Mothers, Anxiety, Depression, Asian Continental Ancestry Group

## Abstract

**Background:**

Accumulating evidence suggests that social support is an important factor with regard to maternal psychological distress. The associations between the contextual factors in terms of social support and the risk of maternal psychological distress have not been adequately studied in Japan. The objective of this study was to examine the association of the presence of advisors on child rearing with maternal anxiety and depressive symptoms among Japanese women at 2 time points after childbirth.

**Methods:**

A self-administered questionnaire that included items regarding the conditions of child rearing and a scale to estimate psychological distress was delivered to 2657 mothers when their infants were 3-4 months and 9-10 months old in 2004-2005. Multivariate logistic regression analysis was conducted for the statistical analyses.

**Results:**

From the multivariate odds ratio, an environment with a few close advisors on child rearing was associated with the risks of maternal anxiety and depressive symptoms at 3-4 months and 9-10 months. The presence of few professional advisors on child rearing was also related to the risk of maternal depressive symptoms at the 2 time periods. The companionship of other child-rearing individuals was related to depressive symptoms at 9-10 months.

**Conclusion:**

An environment without advisors on child rearing was associated with maternal psychological distress. A similarity between the observations at the 2 time points was that the presence of personal and professional advisors was related to maternal anxiety and/or depressive symptoms. It was noted that the need for other child-rearing companions increases as the child grows older.

## INTRODUCTION

The prevalence and characteristics of maternal psychological distress have been widely examined, and its numerous adverse effects on child health have also been reported.^1^^-^^20^ Furthermore, intervention studies aimed at preventing maternal depression and/or anxiety have been conducted.^3^^,^^21^^,^^22^ Most importantly, contextual factors in terms of social support have been recognized to be clearly associated with maternal psychological distress.^18^^,^^23^^-^^28^ Most previous reports on the relationships between contextual factors in terms of social support status and maternal psychological distress have been from Western countries, where the cultural background and the support systems for child-rearing women differ from those in Japan.

In Japan, the national project entitled “Healthy Parents and Children 21” was initiated in 2001.^29^^,^^30^ This project aims to reduce the percentage of women who develop postpartum depression and/or child-rearing anxiety by the year 2010, by increasing the percentage of women who have the support of advisors on child rearing. The percentage of local governments providing some form of child-rearing support in conjunction with the timing of child health checkup services has increased (from 64.4% in 2001 to 89.3% in 2005).^29^ It is noteworthy that an effective support system has been developed on the basis of accumulating evidence on its benefits for child-rearing women. However, this type of evidence is still scant, and to the best of our knowledge, the association between the contextual factors of social support and the risk of maternal psychological distress has not been adequately studied in Japan. The most recent report only examined child-rearing anxiety and the need for improving the social support structure for child-rearing women, and the data were limited to mothers with 1-month-old infants.^31^ In addition, most of the studies conducted to date have methodological limitations with regard to the sample size, area of study, or analysis.^32^

We previously reported the results of a comparison of maternal psychological distress between 2 time-points when the infants were 3-4 months of age and when they were 9-10 months of age among the same participants.^33^ The results suggested that the anxiety symptoms appeared unchanged through the 2 time points, while the scores for depressive symptoms worsened. The objective of this study conducted in Japan was to further examine the associations between the presence of advisors and the risks of maternal anxiety and depressive symptoms at different time points, that is, when their infants were 3-4 months old and 9-10 months old.

## METHODS

### Study Participants

The present study was part of a longitudinal study focused on women’s satisfaction with regard to childbirth and child rearing. The study designs have been described in detail elsewhere.^33^^-^^35^ In brief, prior to their discharge from these centers, women who gave birth in 2004 in any of the 25 collaborating centers were asked to answer a baseline questionnaire that included questions on social background. The collaborating birth centers comprised 4 advanced medical centers (comprehensive prenatal medical center or university hospital), 3 public community hospitals, 5 private clinics, and 13 maternity homes (i.e., houses with a midwife). The collaborating centers were located across 5 prefectures: Hokkaido, Tokyo, Osaka, Hyogo, and Okayama. Women with stillbirth or who gave birth to babies with severe congenital malformations, who were transferred to other hospitals, who had serious medical problems after childbirth, or who were unable to understand Japanese were excluded from the study in advance. A total of 7500 births were recorded, and a total of 2657 women agreed to participate in the series of surveys.

### Survey Conducted during Child Rearing

The following self-administered questionnaire survey was conducted twice: when the infants were 3-4 months old and when they were 9-10 months old. The time points coincide with the time of the infant health checkups, which are planned by many local governments in Japan. The questionnaire included measurements with regard to the conditions of child rearing and the state of maternal psychological distress.

### Characteristics of the Participants

Data collected from the baseline questionnaire included marital status, education, occupation, annual household income, number of family members, number of children, and other relevant information. Obstetric data were collected from the medical records in the collaborating centers after obtaining written consent from the participants, and the birth data were then linked to the data obtained from the returned questionnaires. The obstetric data included information on the mother, including the age at delivery, place of delivery, birth history, and other physical data and information on the infant, including sex, birth weight, and other relevant data.

### Maternal Symptoms of Anxiety and Depression

Self-reported symptoms of psychological distress were measured using a Japanese translation of the original version of the Hospital Anxiety and Depression Scale (HADS).^35^^-^^37^ Screening for psychological distress using HADS is a rapid and less complicated screening method, and both anxiety and depression status can be easily assessed at once. It has been reported that HADS may also be a useful screening instrument for the general population;^38^^-^^40^ moreover, the Japanese version of HADS has been validated in 2 reports.^40^^,^^41^ The article by Hatta et al, who examined the concurrent validity of HADS using the State-Trait Anxiety Inventory (STAI) and Zung’s Self-rating Depression Scale (SDS), reported that the Japanese version of HADS was reliable for use as a screening method for the psychological status of the general population.^40^ The HADS consists of 14 items with response options ranging within a 4-point scale, i.e., from 0 to 3, and it separately measures the levels of anxiety and depression. The total scores may range from 0 (no symptom) to 21 (maximum distress), which means that higher scores indicate a greater degree of each symptom.

### Support from Advisors on Child Rearing

The questions regarding the types of advisors on child rearing (abbreviated to “advisor condition”) were the presence of close advisors, including family and relatives; presence of a professional advisor; and the companionship of other child-rearing individuals. The responses were graded on a scale of 4: many, several, few, and none. The questions were prepared by referring to the Maternal and Child Health Handbook.^42^

### Subjects Lost to Follow-up

Among the enrolled participants, 1688 responded to the survey at 3-4 months (response rate, 64%). Among these, 1408 participants returned to respond to the follow-up survey at 9-10 months (retention rate, 83%). The demographic characteristics of the women who did not respond at 3-4 months and/or at 9-10 months are described in another manuscript.^33^ In brief, certain demographic differences with regard to age, educational background, and income existed between subjects who were followed up successfully and those who were lost to follow-up.

### Statistical Analysis

Data from 1348 participants who provided anxiety and depressive symptom responses to both the surveys, i.e., at 3-4 and at 9-10 months, were analyzed.

The scale for classifying each variable based on the advisor condition was reduced from 4 to 2: enough (many and several) and few (a few and none). This was done because very few participants responded with “many” or “none.”

The thresholds of maternal anxiety and depressive symptoms were 8 or higher in each subscale of HADS. The clinical threshold score for HADS is 11 or higher, although a score of 8 or higher is generally used for research and screening purposes.^43^

Multiple logistic regression analysis was used to calculate the odds ratios (ORs) and 95% confidence intervals (CIs) for anxiety and depressive symptoms relative to the variables for the advisor condition. In these analyses, the following variables were considered to be confounding factors on the basis of the findings of previous studies: the demographic characteristics of the participants including age, presence/absence of a partner, educational background (up to high school, university or college, and graduate school), annual household income (<4.0 million yen, 4.0-8.0 million yen, and >8.0 million yen), number of family members (excluding children, 1 person, 2 persons, and 3 or more persons), number of children (including newborns, 1 child and 2 or more children), place of delivery (comprehensive prenatal medical center, university hospital, public community hospital, private clinic, and maternity home), birth history (primipara or multipara), planned pregnancy (yes or no), infant birth weight (<2500 g or ≥2500 g), and infant health status (good, moderate, and poor). Additionally, other variables of the advisor condition were also included in the analyses.

All statistical analyses were performed using SPSS^®^ 14.0 J for Windows. A two-sided *P* value less than 0.05 was considered statistically significant.

This study was reviewed and approved by the Institutional Review Board of the National Center for Child Health and Development.

## RESULTS

Table 1 shows the distribution of HADS scores among the study participants. The results of percentages compiled using cut-off points were as follows: 7 or lower for the normal level, 8-10 for the possibility of a clinical level, and 11 or higher for a definite clinical level, as described previously.^33^ From the results, we found that women in this survey scored above the cut-off point of 8 in the HADS anxiety scores and HADS depression scores at 3-4 months with percentages of 26% and 19%, respectively. The corresponding percentages at 9-10 months were 26% and 24%, respectively.

**Table 1.** Distributions of Hospital Anxiety and Depression Scale (HADS) scores at 3-4 months and 9-10 months.

Score	At 3-4 months				At 9-10 months			
	
	Anxiety subscale		Depression subscale		Anxiety subscale		Depression subscale	
			
	n	(%)	n	(%)	n	(%)	n	(%)
0	49	(3.6)	41	(3.0)	41	(3.0)	43	(3.2)
1	81	(6.0)	94	(7.0)	66	(4.9)	95	(7.0)
2	125	(9.3)	140	(10.4)	123	(9.1)	120	(8.9)
3	166	(12.3)	182	(13.5)	180	(13.4)	142	(10.5)
4	166	(12.3)	192	(14.2)	153	(11.4)	179	(13.3)
5	170	(12.6)	168	(12.5)	158	(11.7)	172	(12.8)
6	126	(9.3)	140	(10.4)	136	(10.1)	145	(10.8)
7	112	(8.3)	135	(10.0)	139	(10.3)	128	(9.5)
8	87	(6.5)	94	(7.0)	107	(7.9)	119	(8.8)
9	86	(6.4)	61	(4.5)	60	(4.5)	73	(5.4)
10	50	(3.7)	46	(3.4)	42	(3.1)	53	(3.9)
11	47	(3.5)	26	(1.9)	42	(3.1)	35	(2.6)
12	31	(2.3)	6	(0.4)	31	(2.3)	18	(1.3)
13	13	(1.0)	11	(0.8)	25	(1.9)	14	(1.0)
14	14	(1.0)	9	(0.7)	12	(0.9)	5	(0.4)
15	8	(0.6)	2	(0.1)	13	(1.0)	5	(0.4)
16	6	(0.4)	0		9	(0.7)	1	(0.1)
17	4	(0.3)	1	(0.1)	3	(0.2)	0	
18	1	(0.1)	0		4	(0.3)	1	(0.1)
19	2	(0.1)	0		4	(0.3)	0	
20	3	(0.2)	0		0		0	
21	1	(0.1)	0		0		0	

Total	1348	(100)	1348	(100)	1348	(100)	1348	(100)

The participants’ characteristics are shown in Table 2. The total characteristics of the study participants are also briefly described elsewhere.^33^ In short, a majority of the participants were aged 30-39 years (65%), had a partner (98%), and were university or college graduates (76%). Moreover, 68% had an annual household income of 4.0 or more million yen, and 93% had more than 2 family members. With regard to the infants’ characteristics, 92% of the infants had birth weights of ≥2500 g and had a good or moderate health status of 84% at 3-4 months and 74% at 9-10 months. Compared with the participants without anxiety symptoms at 3-4 months, the participants with anxiety symptoms had more than 2 children (*P* = 0.04) and were multiparous (*P* = 0.03), and the infant’s health status was likely to be poor (*P* < 0.001). The corresponding characteristics of the participants with depressive symptoms at 3-4 months were as follows: they were likely to be older (*P* = 0.008) and multiparous (*P* = 0.001) and have more than 2 children (*P* < 0.001), and the infant’s health status was likely to be poor (*P* = 0.008). Compared with the participants without anxiety symptoms at 9-10 months, the participants with anxiety symptoms were multiparous (*P* = 0.02), and the infant’s health status was likely to be poor (*P* = 0.002). The corresponding characteristics of the participants with depressive symptoms at 9-10 months were as follows: they were likely to be older (*P* = 0.01) and multiparous (*P* < 0.001) and have more than 2 children (*P* < 0.001), and the infant’s health status was likely to be poor (*P* < 0.001).

**Table 2.** Characteristics of the participants according to the maternal anxiety and depressive symptom status at 3-4 months and 9-10 months.

Maternal characteristics	Total n = 1348	At 3-4 months						At 9-10 months					
	
		Without Anxiety symptoms*	With Anxiety symptoms*	*P* value^†^	Without Depressive symptoms*	With Depressive symptoms*	*P* value^†^	Without Anxiety symptoms	With Anxiety symptoms*	*P* value^†^	Without Depressive symptoms	With Depressive symptoms*	*P* value^†^
		n = 995	n = 353		n = 1092	n = 256		n = 996	n = 352		n = 1024	n = 324	
Maternal age, year (%)													
<20	8 (100)	5 (63)	3 (38)	0.21	8 (100)	0	0.008	5 (63)	3 (38)	0.28	7 (88)	1 (13)	0.01
20-29	407 (100)	292 (71.7)	115 (28.3)		343 (84.3)	64 (15.7)		290 (71.3)	117 (28.7)		328 (80.6)	79 (19.4)	
30-39	882 (100)	663 (75.2)	219 (24.8)		706 (80)	176 (20)		664 (75.3)	218 (24.7)		655 (74.3)	227 (25.7)	
>39	46 (100)	30 (65)	16 (35)		30 (65)	16 (35)		32 (70)	14 (30)		29 (63)	17 (37)	
Unknown	5 (100)	5 (100)	0		5 (100)	0		5 (100)	0		5 (100)	0	

Partner (%)													
Presence	1329 (100)	979 (73.7)	350 (26.3)	0.19	1076 (81)	253 (19)	0.83	982 (73.9)	347 (26.1)	0.93	1008 (75.8)	321 (24.2)	0.58
Absence	10 (100)	7 (70)	3 (30)		8 (80)	2 (70)		7 (70)	3 (70)		9 (90)	1 (70)	
Unknown	9 (100)	9 (100)	0		8 (89)	1 (11)		7 (70)	2 (22)		7 (78)	2 (22)	

Educational background (%)													
Up to high school	270 (100)	193 (71.5)	77 (28.5)	0.58	226 (83.7)	44 (16.3)	0.39	187 (69.3)	83 (30.7)	0.17	208 (77)	62 (23)	0.89
University or college	1024 (100)	765 (74.7)	259 (25.3)		824 (80.5)	200 (19.5)		771 (75.3)	253 (24.7)		777 (75.9)	247 (24.1)	
Graduate school	44 (100)	30 (68)	14 (32)		33 (70)	11 (25)		30 (68)	14 (32)		32 (73)	12 (70)	
Unknown	10 (100)	7 (70)	3 (70)		9 (90)	1 (70)		8 (70)	2 (70)		7 (70)	3 (30)	

Annual household income, million yen (%)													
<4.0	380 (100)	270 (71.1)	110 (28.9)	0.43	318 (83.7)	62 (16.3)	0.33	272 (71.6)	108 (28.4)	0.64	289 (76.1)	91 (23.9)	0.95
4.0-8.0	547 (100)	409 (74.8)	138 (25.2)		443 (81)	104 (19)		406 (74.2)	141 (25.8)		417 (76.2)	130 (23.8)	
>8.0	378 (100)	286 (75.7)	92 (24.3)		298 (78.8)	80 (21.2)		286 (75.7)	92 (24.3)		287 (75.9)	91 (24.1)	
Unknown	43 (100)	30 (70)	13 (30)		33 (77)	10 (23)		32 (70)	11 (26)		31 (72)	12 (70)	

Number of family members, excluding children (%)													
1	80 (100)	52 (65)	28 (35)	0.07	62 (78)	18 (23)	0.08	51 (64)	29 (36)	0.05	60 (75)	20 (25)	0.45
2	1187 (100)	883 (74.4)	304 (25.6)		969 (81.6)	218 (18.4)		881 (74.2)	306 (25.8)		906 (76.3)	281 (23.7)	
≥3	72 (100)	51 (71)	21 (29)		52 (72)	20 (28)		55 (76)	17 (24)		50 (69)	22 (31)	
Unknown	9 (100)	9 (100)	0		9 (100)	0		9 (100)	0		8 (89)	1 (11)	

Number of children, including newborns (%)													
1	657 (100)	503 (76.6)	154 (23.4)	0.04	561 (85.4)	96 (14.6)	< 0.001	506 (77)	151 (23)	0.20	535 (81.4)	122 (18.6)	< 0.001
≥2	688 (100)	489 (71.1)	199 (28.9)		529 (76.9)	159 (23.1)		487 (70.8)	201 (29.2)		487 (70.8)	201 (29.2)	
Unknown	3 (100)	3 (100)	0		2 (67)	1 (33)		3 (100)	0		2 (67)	1 (33)	

Place of delivery (%)													
Comprehensive perinatal medical center	647 (100)	464 (71.7)	183 (28.3)	0.26	504 (77.9)	143 (22.1)	0.02	462 (71.4)	185 (28.6)	0.29	480 (74.2)	167 (25.8)	0.11
University hospital	24 (100)	16 (67)	8 (33)		18 (75)	6 (25)		20 (83)	4 (17)		19 (79)	5 (21)	
Public community hospital	67 (100)	55 (82)	12 (18)		61 (91)	6 (9)		52 (78)	15 (22)		56 (84)	11 (16)	
Private clinic	196 (100)	147 (75)	49 (25)		162 (82.7)	34 (17.3)		146 (74.5)	50 (25.5)		141 (71.9)	55 (28.1)	
Maternity home	414 (100)	313 (75.6)	101 (24.4)		347 (83.8)	67 (16.2)		316 (76.3)	98 (23.7)		328 (79.2)	86 (20.8)	

Birth history (%)													
Primipara	631 (100)	483 (76.5)	148 (23.5)	0.03	538 (85.3)	93 (14.7)	0.001	488 (77.3)	143 (22.7)	0.02	516 (81.8)	115 (18.2)	< 0.001
Multipara	701 (100)	498 (71)	203 (29)		541 (77.2)	160 (22.8)		495 (70.6)	206 (29.4)		496 (70.8)	205 (29.2)	
Unknown	16 (100)	14 (88)	2 (13)		13 (81)	3 (19)		13 (81)	3 (19)		12 (75)	4 (25)	

Planned pregnancy (%)													
Yes	1238 (100)	918 (74.2)	320 (25.8)	0.50	1003 (81)	235 (19)	0.89	924 (74.6)	314 (25.4)	0.08	942 (76.1)	296 (23.9)	0.78
No	109 (100)	76 (69.7)	33 (30.3)		88 (80.7)	21 (19.3)		71 (65.1)	38 (34.9)		81 (74.3)	28 (25.7)	
Unknown	1 (100)	1 (100)	0		1 (100)	0		1 (100)	0		1 (100)	0	

Infant characteristics													
Infant sex (%)													
Female	684 (100)	502 (73.4)	182 (26.6)	0.76	556 (81.3)	128 (18.7)	0.84	502 (73.4)	182 (26.6)	0.71	517 (75.6)	167 (24.4)	0.75
Male	664 (100)	493 (74.2)	171 (25.8)		536 (80.7)	128 (19.3)		494 (74.4)	170 (25.6)		507 (76.4)	157 (23.6)	

Birth weight, g (%)													
<2500	96 (100)	67 (70)	29 (30)	0.40	75 (78)	21 (22)	0.50	73 (76)	23 (24)	0.63	75 (78)	21 (22)	0.71
≥2500	1252 (100)	928 (74.1)	324 (25.9)		1017 (81.2)	235 (18.8)		923 (73.7)	329 (26.3)		949 (75.8)	303 (24.2)	

Variables related to child rearing ^‡^													
Infant’s health status at 3-4 months (%)													
Good	362 (100)	293 (80.9)	69 (19.1)	< 0.001	310 (85.6)	52 (14.4)	0.008	**- -**	**- -**		**- -**	**- -**	
Moderate	782 (100)	575 (73.5)	207 (26.5)		631 (80.7)	151 (19.3)		**- -**	**- -**		**- -**	**- -**	
Poor	198 (100)	122 (61.6)	76 (38.4)		146 (73.7)	52 (26.3)		**- -**	**- -**		**- -**	**- -**	
Unknown	6 (100)	5 (83)	1 (17)		5 (83)	1 (17)		**- -**	**- -**		**- -**	**- -**	

Infant’s health status at 9-10 months (%)													
Good	359 (100)	**- -**	**- -**		**- -**	**- -**		286 (79.7)	73 (20.3)	0.002	304 (84.7)	55 (15.3)	< 0.001
Moderate	647 (100)	**- -**	**- -**		**- -**	**- -**		475 (73.4)	172 (26.6)		475 (73.4)	172 (26.6)	
Poor	320 (100)	**- -**	**- -**		**- -**	**- -**		216 (67.5)	104 (32.5)		225 (70.3)	95 (29.7)	
Unknown	22 (100)	**- -**	**- -**		**- -**	**- -**		19 (86)	3 (14)		20 (91)	2 (9)	

*: A Hospital Anxiety and Depression Scale (HADS) score of <7 reflects cases without the symptoms, and a score ≥8 reflects cases with the symptoms.

†: *P* values were calculated by the chi-square test.

‡: Data were obtained at the time of each survey.

Table 3 shows the results of the logistic regression analysis. With regard to the crude ORs, all the types of advisors on child rearing were associated with maternal anxiety symptoms as well as depressive symptoms in both the survey periods. The multivariate logistic regression analysis showed a statistically significant association between few close advisors with both anxiety and depressive symptoms at 3-4 months, with corresponding ORs of 3.46 and 2.50. There was also a statistically significant association between few professional advisors and the risk of depressive symptoms at 3-4 months, with an OR of 1.63. The survey at 9-10 months showed a statistically significant association between few close advisors with both anxiety and depressive symptoms, with corresponding ORs of 1.84 and 1.78. Few professional advisors and few companions were also statistically significantly associated with the risk of depressive symptoms, with ORs of 1.55 and 1.76, respectively.

**Table 3.** Association between advisor condition and both maternal anxiety and depressive symptom status at 3-4 months and 9-10 months.

Variables of the advisor condition	n	Anxiety symptoms^†^			Depressive symptoms^†^		
	
		Number of Cases with anxiety symptoms (%)	Crude odds ratio (95% CI)^¶^	Adjusted odds ratio (95% Cl)^§^	Number of Cases with depressive symptoms (%)	Crude odds ratio (95% CI)^¶^	Adjusted odds ratio (95% Cl)^§^
At 3-4 months							
Close advisors on child rearing							
Enough	1233	290 (23.5)	1.00 (reference)	1.00 (reference)	210 (17.0)	1.00 (reference)	1.00 (reference)
Few	115	63 (54.8)	3.94 (2.67-5.82)***	3.46 (2.19-5.48)***	46 (40.0)	3.25 (2.17-4.85)***	2.50 (1.54-4.05)***

Professional advisors on child rearing							
Enough	887	210 (23.7)	1.00 (reference)	1.00 (reference)	141 (15.9)	1.00 (reference)	1.00 (reference)
Few	459	142 (30.9)	1.44 (1.12-1.86)**	1.23 (0.94-1.63)	114 (24.8)	1.75 (1.32-2.31)***	1.63 (1.19-2.20)**

Companionship of other child-rearing individuals							
Enough	1237	306 (24.7)	1.00 (reference)	1.00 (reference)	221 (17.9)	1.00 (reference)	1.00 (reference)
Few	110	47 (42.7)	2.27 (1.52-3.38)***	1.42 (0.88-2.31)	35 (31.8)	2.15 (1.40-3.29)***	1.50 (0.89-2.55)

At 9-10 months							
Close advisors on child rearing							
Enough	1238	304 (24.6)	1.00 (reference)	1.00 (reference)	275 (22.2)	1.00 (reference)	1.00 (reference)
Few	110	48 ( 43.6)	2.38 (1.60-3.54)***	1.84 (1.09-3.09)*	49 (44.5)	2.81 (1.89-4.19)***	1.78 (1.04-3.04)*

Professional advisors on child rearing							
Enough	924	221 (23.9)	1.00 (reference)	1.00 (reference)	191 (20.7)	1.00 (reference)	1.00 (reference)
Few	418	130 (31.1 )	1.44 (1.11-1.86)**	1.23 (0.93-1.63)	131 (31.3)	1.75 (1.35-2.27)***	1.55 (1.16-2.08)**

Companionship of other child-rearing individuals							
Enough	1236	307 (24.8)	1.00 (reference)	1.00 (reference)	276 (22.3)	1.00 (reference)	1.00 (reference)
Few	110	45 (40.9)	2.09 (1.40-3.13)***	1.38 (0.82-2.33)	47 (42.7)	2.59 (1.74-3.87)***	1.76 (1.03-3.02)*

†: A Hospital Anxiety and Depression Scale (HADS) score ≥8 for each anxiety and depressive subscale reflects cases with the symptoms at the each survey period.

‡: Variables were collected at each survey month. Participants who did not respond to each question are not included in the table.

§: Adjusted for the following variables: maternal age, presence of a partner, maternal educational background, annual household income, number of family members, number of children, place of delivery, birth history, planned pregnancy, infant birth weight, infant health status, and other variables in the table.

*: *P* < 0.05, **: *P* < 0.01, ***: *P* < 0.001

¶CI: confidence interval

We attempted to conduct a stratified analysis according to maternal age, number of children, birth history, and infant health status because each of these factors differed significantly among the normal cases and cases of anxiety and/or depressive symptoms. Association between each variable of the advisor condition and the risk of anxiety and/or depressive symptoms was consistently observed regardless of maternal age, i.e., at 20-29 or 30-39 years, regardless of the number of children, parity, and infant health status.

## DISCUSSION

This is the latest report that examines the association of contextual factors with respect to support conditions, especially the existence of advisors on child rearing, with the risks of anxiety and depressive symptoms at 2 different child-rearing periods among Japanese women. In this study, an environment with few advisors was positively associated with the risk of psychological distress among child-rearing women in Japan. Overall, multivariate analysis revealed that a close advisor and a professional advisor are required at both 3-4 months and 9-10 months, while the companionship of other child-rearing individuals is required more at 9-10 months than at 3-4 months.

Numerous studies have been conducted on maternal psychological distress, though less attention has been paid to maternal anxiety than to maternal depression.^8^^,^^18^ Meanwhile, several studies have reported an association between maternal anxiety and child health, which is also a predictor of later maternal depression.^2^^,^^3^^,^^8^^,^^17^ Our previous report revealed that the percentage of maternal anxiety symptoms was higher than that of maternal depressive symptoms at both 3-4 months and 9-10 months after childbirth.^33^ Studies have examined both anxiety and depressive symptoms, but further examination of the related environmental factors was limited.^8^ Our study included measures of both anxiety and depressive symptoms. On the basis of the present results, the presence of fewer close advisors was more strongly related to anxiety symptoms than depressive symptoms (OR at 3-4 months, 3.46 and 2.50 respectively). The presence of a person who can give advice as well as engage in casual banter is an important factor in easing maternal anxiety, even though lack of close advisors was substantially related to depressive symptoms. Lack of professional advisors and less companionship were also associated with maternal depressive symptoms. Mothers with less social support tended to suffer from strong psychological distress because they had to cope alone with the infants’ demands.^24^ The effect of interpersonal relationships on maternal depressive symptoms was suggested in another study.^18^^,^^26^

The strengths of the present study are as follows. Previous studies had a limited sample size or the study area was restricted, and the physical condition of the participants was not adequately considered.^32^ The survey in the current study was conducted with the participation of several medical centers, which resulted in the inclusion of a large number of participants, and HADS was also used to estimate psychological symptoms and thereby avoid the confounding effects of hidden physical conditions.^33^^-^^35^

This study also has some limitations. First, while we adjusted the data using measured potential confounders, the effects of unmeasured confounders could not be completely excluded because various situations might influence the development of psychological symptoms in the mother.^9^^,^^23^ Some researchers have reported that women who have a history of depression and anxiety before or during pregnancy, less social support, and experienced distressing life events during pregnancy were likely to have postpartum depressive symptoms.^28^^,^^44^^,^^45^ We could not collect this information. Second, 47% of the participants failed to respond to both the surveys. The reasons for refusal to participate in the survey at 3-4 months and/or 9-10 months were not investigated.

In conclusion, this study found that an environment with few advisors on child rearing or few child-rearing companions tended to be strongly associated with the risk of maternal psychological distress in Japan. Further evidence is required to develop recommendations for ensuring effective social support for child-rearing women.
